# Reduced Impact of Endovascular Thrombectomy on Disability in Real-World Practice, Relative to Randomized Controlled Trial Evidence in Australia

**DOI:** 10.3389/fneur.2020.593238

**Published:** 2020-12-08

**Authors:** Lan Gao, Elise Tan, Marj Moodie, Mark Parsons, Neil J. Spratt, Christopher Levi, Kenneth Butcher, Timothy Kleinig, Bernard Yan, Chushuang Chen, Longting Lin, Philip Choi, Andrew Bivard

**Affiliations:** ^1^Deakin Health Economics, Institute for Health Transformation, Deakin University, Geelong, VIC, Australia; ^2^Department of Neurology, University of New South Wales (UNSW) South Western Clinical School, Liverpool Hospital, University of New South Wales, Liverpool, NSW, Australia; ^3^Departments of Neurology, John Hunter Hospital, University of Newcastle, Newcastle, NSW, Australia; ^4^Melbourne Brain Centre, Royal Melbourne Hospital, Parkville, VIC, Australia; ^5^Prince of Wales Clinical School, University of New South Wales, Sydney, NSW, Australia; ^6^Department of Neurology, Royal Adelaide Hospital, Adelaide, SA, Australia; ^7^School of Medicine and Public Health, University of Newcastle, Newcastle, NSW, Australia; ^8^Department Neuroscience, Eastern Health, Eastern Health Clinical School, Monash University, Melbourne, VIC, Australia

**Keywords:** thrombectomy, disability adjusted life year (DALY), INSPIRE registry, real-world data analysis, randomized controlled clinical trial (RCT)

## Abstract

**Background and Aims:** Disability-adjusted life years (DALYs) are an important measure of the global burden of disease that informs patient outcomes and policy decision-making. Our study aimed to compare the DALYs saved by endovascular thrombectomy (EVT) in the Australasian-based EXTEND-IA trial vs. clinical registry data from EVT in Australian routine clinical practice.

**Methods:** The 3-month modified Rankin scale (mRS) outcome and treatment status of consecutively enrolled Australian patients with large vessel occlusion (LVO) stroke were taken from the International Stroke Perfusion Imaging Registry (INSPIRE). DALYs were calculated as the summation of years of life lost (YLL) due to premature death and years lived with a disability (YLD). A generalized linear model (GLM) with gamma family and log link was used to compare the difference in DALYs for patients receiving/not receiving EVT while controlling for key covariates. Ordered logit regression model was utilized to compare the difference in functional outcome at 3 months between the treatment groups. Cox regression analysis was undertaken to compare the difference in survival over an 18-year time horizon. Estimated long-term DALYs saved based on the EXTEND-IA randomized controlled trial (RCT) results were used as the comparator.

**Results:** INSPIRE patients who received EVT treatment only achieved nominally better functional outcomes than the non-EVT group (*p* = 0.181) at 3 months. There was no significant survival gain from EVT over the first 3 months of stroke in both INSPIRE and EXTEND-IA patients. However, measured against no EVT in the long-term, EVT in INSPIRE was associated with no significant survival gain [hazard ratio (HR): 0.92, 95% confidence interval (CI): 0.78–1.08, *p* = 0.287] compared with the survival benefit extrapolated from the EXTEND-IA trial (HR: 0.42, 95% CI: 0.22–0.82, *p* = 0.01]. Offering EVT to patients with LVO stroke was also associated with fewer DALYs lost (11.04, 95% CI: 10.45–11.62) than those not receiving EVT in INSPIRE (12.13, 95% CI: 11.75–12.51), a reduction of −1.09 DALY (95% CI: −1.76 to −0.43, *p* = 0.002). The absolute magnitude of the treatment effect was lower than that seen in EXTEND-IA (−2.72 DALY reduction in EVT vs non-EVT patients).

**Conclusions:** EVT for the treatment of LVO in a registry of routine care was associated with significantly lower DALYs lost than medical care alone, but the saved DALYs are less than those reported in clinical trials, as there were major differences in the baseline characteristics of the patients.

## Introduction

Endovascular thrombectomy (EVT) is associated with significantly improved disability-free survival compared with the previous standard of care, intravenous thrombolysis (IVT) (1–6). One of these positive normal time window EVT trials, EXTEND-IA, in which selection by perfusion imaging demonstration of “target mismatch” was mandatory, reported that EVT led to a doubling of the number of people living disability free (generalized odds ratio (OR) 2.0, 95% confidence interval (CI): 1.2–3.8, *p* = 0.006 on ordinal analysis of modified Rankin scale (mRS) score) (6). However, analysis based on routine clinical practice following implementation of trial evidence is not always the same as that observed in randomized controlled trials (RCTs). Patients may be treated outside of the strict trial criteria. For example, most trials of EVT had an age limit for inclusion. In a real-world study, elderly patients who received EVT experienced higher rates of hemorrhage (40.7 vs. 9.3%, *p* < 0.001) without significantly improved functional outcomes than a matched medical management cohort (7). A previous study that examined the Disability-adjusted life years (DALYs) lost based on the EXTEND-IA RCT, which utilized perfusion imaging selection to identify patients with large vessel occlusion (LVO) stroke for EVT, reported that EVT led to significantly fewer lifetime DALYs lost than non-EVT (−2.72 DALY, *p* = 0.02) (8). However, DALY estimation from this study may not be generalizable to real-world practice as perfusion imaging selection (or the criteria if performed) for EVT is not rigidly adhered to (or not performed at all in some countries).

DALYs were developed to measure the relative and total global burden of all diseases using a common metric, which integrates both mortality (years of life lost (YLL) due to premature death) and morbidity (years of healthy life lost due to living with a disability) (9). As an important metric of disease burden, the DALYs can be used as the basis for resource allocation and priority setting for stroke prevention, evidence-based healthcare planning for acute stroke management, and post-stroke rehabilitation services (10). They have been referenced extensively in global health debates and decision-making (11). DALYs can reveal the likely impact of stroke on a patient over their remaining lifetime rather than at a single time point. Stroke, as the leading cause of adult disability in Australia and worldwide, leads to significantly more DALYs lost (116.4 million) annually than coronary heart disease (82 million) (12, 13).

Even though there is controversy around the use of DALYs as an outcome measure (14), they remain an important index to quantify the disease burden (15). Given the important role that DALYs play in informing policy decision-making, this study aims to improve the evidence based on comparing the DALYs saved from EVT in LVO stroke patients with LVO to assess the impact of the implementation of EVT into routine clinical care compared with the benefits reported in EXTEND-IA in Australia. Other important clinical outcomes including 3-month mRS score and overall survival were also analyzed and compared.

## Methods

### Study Population

We accessed the International Stroke Perfusion Imaging Registry (INSPIRE), a registry of patients with stroke, to source the baseline and 90-day clinical and imaging data for all Australian patients with LVO defined as occlusions of the internal carotid artery (ICA) and proximal segment (M1, M2) of the middle cerebral artery over a 5-year period (2015–2019), regardless of the baseline characteristics (e.g., volume of infarct core, etc.) (16, 17). Routine multimodal computed tomography (CT: non-contrast CT, perfusion CT, and CT angiography) was performed on patients presenting with acute neurological deficits. Eligible patients received thrombolysis prior to EVT. In routine clinical practice, EVT is offered to patients with (i) the presence of a LVO that is potentially retrievable, (ii) reasonable pre-stroke function (mRS 0–3), and (iii) limited medical comorbidities. In particular, if the patient was within 6 h of onset, perfusion imaging criteria, such as used in EXTEND-IA, was not used to exclude patients from therapy. Unless contraindicated, magnetic resonance imaging (MRI) at 24 h post-stroke was standard practice, in which case repeat imaging was carried out with multimodal CT. The National Institute of Health Stroke Severity (NIHSS), the primary index for stroke severity, was assessed immediately before treatment and 24 h imaging. In INSPIRE, 24-h Thrombolysis in Cerebral Infarction (TICI) Score is adopted as reperfusion grades with 2b−3 being successful reperfusion (18). All patients were assessed at 90 days post-stroke onset for functional outcome using the mRS.

Written informed consent for participation in the study was provided by all the patients. The Hunter New England Area Health Service Human Research Ethics Committee reviewed and approved the study protocol in 2012.

### Estimation of DALY

A DALY is calculated as the summation of two components: YLL due to premature death and years lived with a disability (YLD). Lower DALYs indicate more disability-free life years after the index stroke (one DALY indicates 1 year of disability-free life lost).

YLL due to premature death are calculated as the difference in life expectancy of an age-, sex-matched person from the general Australian population and a stroke patient in a certain mRS category. Patients post-stroke have a higher hazard ratio (HR) of dying from all-cause mortality determined by their mRS score at 3 months. All-cause mortality of the general Australian population for each age and gender was adjusted by the corresponding HR to estimate the life expectancy for patients with stroke (19). The life table for the general Australian population and the formula to recalculate the life expectancy for stroke patients were sourced from the Australian Bureau of Statistics (ABS) (20).

YLD are calculated by multiplying the life expectancy of a stroke patient by a disability weight. The disability weights based on mRS score were sourced from the study by Hong and Saver (21). In the base case, DALYs were not adjusted by age weight nor discounted, in accordance with the recommendation of the World Health Organization (19). However, in the sensitivity analysis, DALYs were discounted at an annual rate of 3%, and age weighting was applied to examine the robustness of base case results (19). Using individual patient-level data from INSPIRE, DALYs (and the two components—YLL and YLD) were calculated for each patient based on their 3-month mRS score. The detailed calculations are provided in the Supplementary Document.

### Statistical Analysis

Descriptive analysis was undertaken of the baseline characteristics of Australian patients receiving EVT vs. no EVT using INSPIRE data and compared with their respective groups in the Australasian-based EXTEND-IA trial. EXTEND-IA was selected as the comparator since it offers the optimal comparability of EVT/no EVT patients with INSPIRE, by reporting the long-term DALY outcome using patient-level data and comparing patients treated in the same centers and similar medical systems by the same healthcare providers across the country. Differences in baseline characteristics within INSPIRE and between INSPIRE and EXTEND-IA were tested using *T*-test or Wilcoxon rank-sum test depending on the distribution of the variable (22). Within INSPIRE, (i) ordered logit regression model was utilized to compare the difference in functional outcome at 3 months between the treatment groups (23); (ii) Cox regression model (survival analysis) was undertaken to explore the difference in the projected survival over 18 years (from 3-month post-stroke onset) between the two treatment groups (8). These analyses were also performed in a subgroup of patients with intracranial ICA or M1 occlusions as this is the group with the strongest evidence of benefit from thrombectomy. Eighteen years were considered sufficient to capture the difference in survival in the current study given the older age of many stroke patients. The time-to-death for each individual was extracted from the reconstructed life table for patients with stroke but was censored at 18 years post the index stroke. Where a patient died due to stroke (mRS = 6) prior to month 3, they were assigned the maximal survival of 3 months. In the Cox regression model, onset age, gender, baseline NIHSS, baseline infarct core volume, and baseline penumbra volume were adjusted, which are in line with the analysis from EXTEND-IA. Survival gain and DALYs saved due to EVT within INSPIRE were compared with similar outcomes (i.e., survival and DALYs saved from both EVT and no EVT treatment groups) estimated from EXTEND-IA (6).

To estimate the difference in DALYs, YLL and YLD between the two treatment groups, a generalized linear model (GLM) with gamma distribution and log link was adopted given the non-normal distribution of the DALY and its two components. The selection of gamma distribution and link function was informed by a modified Park test and link test (24). DALY was the dependent variable, whereas the treatment status, onset age, gender (male/female), time from stroke onset to hospital arrival (min), baseline NIHSS, baseline penumbra volume, and IVT treatment (Y/N) were entered as covariates. DALYs saved (i.e., between-group difference in DALYs) due to EVT from INSPIRE and EXTEND-IA were directly compared. To further test the difference in DALYs lost within INSPIRE by age, onset age was also coded as a dummy variable and was included in the GLM analysis. Bootstrapping with 2,000 iterations was undertaken to further examine the robustness of results from GLM analysis. All the analyses were performed using the STATA V16 statistical package (StataCorp 2019, Stata Statistical Software: Release 16; StataCorp LLC, College Station, TX, USA) and Microsoft Excel.

## Results

### Study Populations

In total, from INSPIRE, 178 LVO patients (mean age of 69.7 years, 53% male) received EVT vs. 584 LVO patients (mean age of 71.9 years, 52% male) who had medical treatment alone during the study period (2015–19). The age distribution of patients from the two treatment groups is shown in Supplementary Figure 1. The median baseline NIHSS was 16 and 15 in the two groups with similar interquartile ranges (IQRs). The baseline core volume was not significantly different between the two groups, whereas patients who received EVT had significantly higher penumbra volume at baseline (88 ml in the EVT group vs. 68 ml in the non-EVT group, *p* < 0.001). Approximately 48.3% of patients in the EVT group had thrombolysis prior to EVT compared with 59.3% in the non-EVT group, and 83% of patients received EVT within 6 h of stroke onset. Patients who received EVT treatment only achieved nominally better functional outcomes than the non-EVT group (*p* = 0.181) at 3 months and when controlled for both time from stroke onset to hospital arrival and successful reperfusion at 24 h (i.e., TICI 2b/3), and the results were consistent (*p* = 0.382). Baseline characteristics and 3-month mRS outcomes of the study population are summarized in Table 1 and Supplementary Figure 2. Three-month mRS outcomes for subgroups with ICA or M1 occlusions, provided in Supplementary Table 1, show that EVT was associated with significantly better functional outcomes than patients not receiving EVT (*p* = 0.017).

**Table 1:** Baseline characteristics of the LVO study population from INSPIRE and EXTEND-IA.

	**INSPIRE**		**EXTEND-IA RCT**	
	**EVT**	**No EVT**	**EVT**	**No EVT**
	**(*****N*** **= 178)**	**(*****N*** **= 584)**	**(*****N*** **= 35)**	**(*****N*** **= 35)**
Age (mean, SD)	69.7 (14.76)	71.9 (13.52)	68.6 (12.3)	70.2 (11.8)
Male gender (*N*, %)	95 (53.4%)	301 (51.5%)	17 (49%)	17 (49%)
Baseline NIHSS (median, IQR)	16 (11–21)	15 (10–18)	17 (13–20)	13 (9–19)
Baseline core volume (median, IQR)	18 (7–36)	18 (7–40)	12 (4–32)	18 (4–29)
Perfusion lesion volume (median, IQR)	112 (74–151)	83 (49–133)	106 (76–137)	115 (72–158)
**Treatment type (*****N*****, %)**				
EVT + tPA	86 (48.3%)	0	35 (100%)^*^	0
EVT only	92 (51.7%)	0	0	0
tPA only	0	346 (59.3%)	0	35 (100%)
Target mismatch	156 (87.6%)	452 (77.4%)	100%	100%
Core volume ≥70 ml	11 (6.2%)	68 (11.6%)	0%	0%
Median time from onset to hospital arrival	95 (43–148)	112 (53–171)	78 (54–112)	80 (56–115)
Median time from onset to tPA	156 (120–187)	171 (127–211)	127 (93–162)	145 (105–180)
Successful reperfusion (*N*, %)	90 (74.4%)^**^	242 (53.7%)^**^	35 (100%)	13 (37%)
**Occlusion site (*****N*****, %)**				
ICA	53 (29.8%)	151 (25.9%)	11 (31%)	11 (31%)
M1	115 (64.6%)	310 (53.1%)	20 (57%)	18 (51%)
M2	10 (5.6%)	123 (21.0%)	4 (11%)	6 (17%)
**Three-month mRS**				
mRS 0	27 (15.2%)	93 (15.9%)	9 (26%)	6 (17%)
mRS 1	39 (21.9%)	96 (16.4%)	9 (26%)	4 (11%)
mRS 2	19 (10.7%)	68 (11.6%)	7 (20%)	4 (11%)
mRS 3	41 (23.0%)	76 (13.0%)	6 (17%)	4 (11%)
mRS 4	15 (8.4%)	85 (14.6%)	1 (3%)	6 (17%)
mRS 5	8 (4.5%)	60 (10.3%)	0 (0%)	4 (11%)
mRS 6	29 (16.3%)	106 (18.2%)	3 (9%)	7 (20%)

*Age, gender, baseline NIHSS, volumes of infarct core and perfusion lesion, and occlusion locations are not significantly different for patients who received EVT between INSPIRE and EXTEND-IA participants*.

* On the basis of intention-to-treat principle;

** *57 patients in the EVT and 133 patients in the no EVT group did not have the 24-h TICI outcome. The proportion of successful reperfusion was significantly different between the EVT and no EVT groups within INSPIRE (p < 0.0001)*.

*Baseline core volume, perfusion lesion volume, and penumbra volume are expressed in ml*.

*EVT, endovascular thrombectomy; IQR, interquartile range; SD, standard deviation; ICA, internal carotid artery; tPA, tissue plasminogen activator*.

In comparison, the EXTEND-IA RCT recruited patients with a mean age of 69 years (SD = 12), median NIHSS score of 15 (IQR: 12–19), and 49% males (6). The median baseline core and perfusion lesion volumes were 12 vs. 18 ml and 106 vs. 115 ml in the EVT and no EVT treatment groups, respectively. Generally, patients receiving EVT in both INSPIRE and EXTEND-IA were comparable in terms of some key baseline characteristics. However, there were proportionally less patients with M2 occlusions offered EVT, higher proportion with large infarct core (thus smaller proportion with target mismatch), and longer time from stroke onset to hospital arrival in INSPIRE.

### Survival Analysis

**Figure 1:**
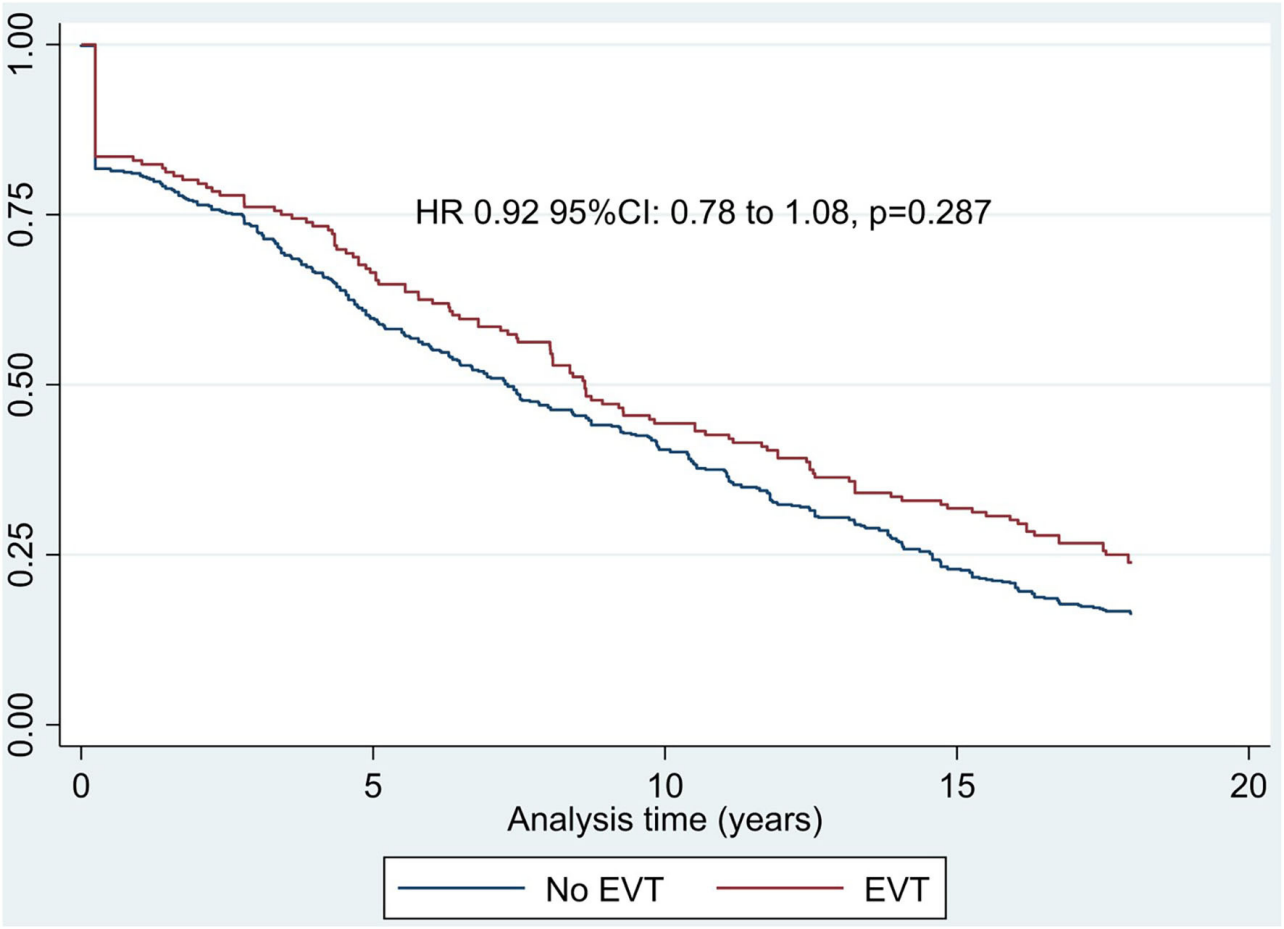
Cox proportional hazards regression for the long-term modeled survival.

Based on the published HR of mortality by mRS score for stroke patients, the Cox regression model showed that INSPIRE EVT was associated with nominally reduced probability of death (HR: 0.92, 95% CI: 0.78–1.08, *p* = 0.287, Figure 1). Older age, being male, and higher baseline volume of baseline infarct core contributed to decreased probability of survival (*p* < 0.05), whereas baseline NIHSS (*p* = 0.146) was not statistically significant for the probability of survival (Supplementary Table 2). The subgroup analysis including patients with ICA or M1 occlusions suggested that EVT led to a reduced probability of mortality (HR: 0.82, 95% CI: 0.71–0.96, *p* = 0.012) with older age, male gender, and higher baseline NIHSS accounting for lowered probability of survival (Supplementary Figure 3).

The long-term estimation based on the EXTEND-IA data reported that EVT was associated with a better probability of survival with a HR of 0.42 (95% CI: 0.22–0.82, *p* = 0.01) than corresponding controls with no EVT (8).

### DALYs Lost

EVT in INSPIRE LVO patients was associated with fewer DALYs lost (11.04, 95% CI: 10.45–11.62) than those not receiving EVT (12.13, 95% CI: 11.75–12.51) or a difference in reduction of 1.09 DALYs (95% CI: −1.76 to −0.43, *p* = 0.002). Similar results were also observed for YLL and YLD, with corresponding avoided YLL of −0.68 (95% CI: −1.18−0.18, *p* = 0.009) and YLD of −0.47 (95% CI: −0.98 to 0.03, *p* = 0.074). The raw distribution of DALYs by treatment groups is presented in Supplementary Figure 4. In the scenario where the age weighting and discounting were applied, the difference in DALYs saved was smaller (as future life years were discounted) (Table 2). The unadjusted difference in EXTEND-IA DALYs was 2.72 (*p* = 0.02) (8). In summary, the between-group difference in DALYs reduction due to EVT was less in the INSPIRE population (1.09 DALY saved) than that in the EXTEND-IA RCT (2.72 DALY saved).

**Table 2:** Results of disability-adjusted life years by endovascular thrombectomy status from INSPIRE and EXTEND-IA.

	**INSPIRE**				**EXTEND-IA RCT**			
	**YLL**		**YLD**		**DALY**		**DALY**	
	**Unadjusted**	**Adjusted**	**Unadjusted**	**Adjusted**	**Unadjusted**	**Adjusted**	**Unadjusted**	**Adjusted**
EVT	8.77 (8.35, 9.20)	2.61 (2.36, 2.86)	2.66 (2.14, 3.18)	1.74 (1.36, 2.13)	11.04 (10.45, 11.62)	3.96 (3.64, 4.27)	5.5 (3.2, 8.7)	2.0 (0.94, 3.6)
No EVT	9.45 (9.19, 9.72)	2.98 (2.82, 3.14)	3.13 (2.67, 3.60)	2.10 (1.72, 2.47)	12.13 (11.75, 12.51)	4.59 (4.37, 4.81)	8.9 (4.7, 13.8)	3.5 (1.6, 5.8)
Between-group difference	−0.68 (−1.18, −0.18)	−0.37 (−0.67, −0.07)	−0.47 (−0.98, 0.03)^*^	−0.35 (−0.70, −0.01)	−1.09 (−1.76, −0.43)	−0.63 (−0.99, −0.27)	−2.72 (N.A.)	−1.27 (N.A.)

*EVT, endovascular thrombectomy; YLL, years of life lost; YLD, years lived with a disability; DALY, disability-adjusted life year; N.A., not available*.

* *Not statistically significant (p = 0.074)*.

### Sensitivity Analysis

**Figure 2:**
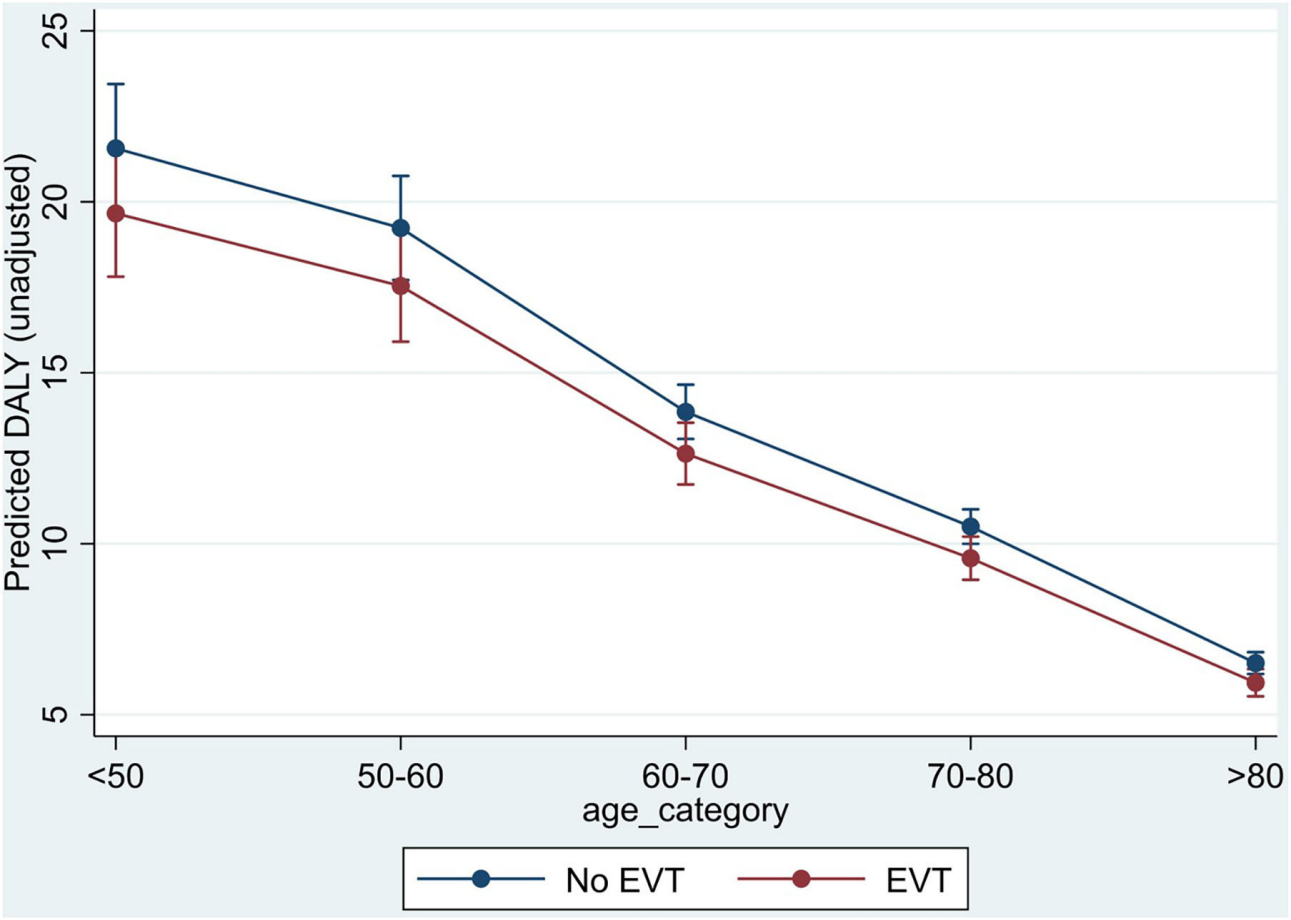
Difference in disability-adjusted life years by age category.

The analysis by age group showed that in the INSPIRE population, DALYs saved decreased by age due to the lesser impact on remaining life expectancy (Figure 2). The larger difference in DALYs saved −1.90 (95% CI: −3.15 to −0.65) was observed in the youngest age group (<50 years), whereas this difference reduced to −0.57 (95% CI: −0.95 to −0.20) in the oldest age category (>80 years) (Table 3).

**Table 3:** Results of disability-adjusted life years by age category from INSPIRE.

**Age**	**EVT_INSPIRE**	**No EVT_INSPIRE**	**Between-group**
**category**			**difference (DALY saved)**
<50	19.66 (17.81, 21.51)	21.56 (19.68, 23.44)	−1.90 (−3.15, −0.65)
50–60	17.54 (15.91, 19.17)	19.23 (17.71, 20.76)	−1.70 (−2.80, −0.60)
60–70	12.64 (11.73, 13.54)	13.86 (13.06, 14.65)	−1.22 (−2.02, −0.43)
70–80	9.58 (8.95, 10.21)	10.50 (10.00, 11.01)	−0.93 (−1.53, −0.33)
>80	5.94 (5.54, 6.33)	6.51 (6.19, 6.83)	−0.57 (−0.95, −0.20)

*EVT, endovascular thrombectomy; DALY, disability-adjusted life year*.

**Figure 3:**
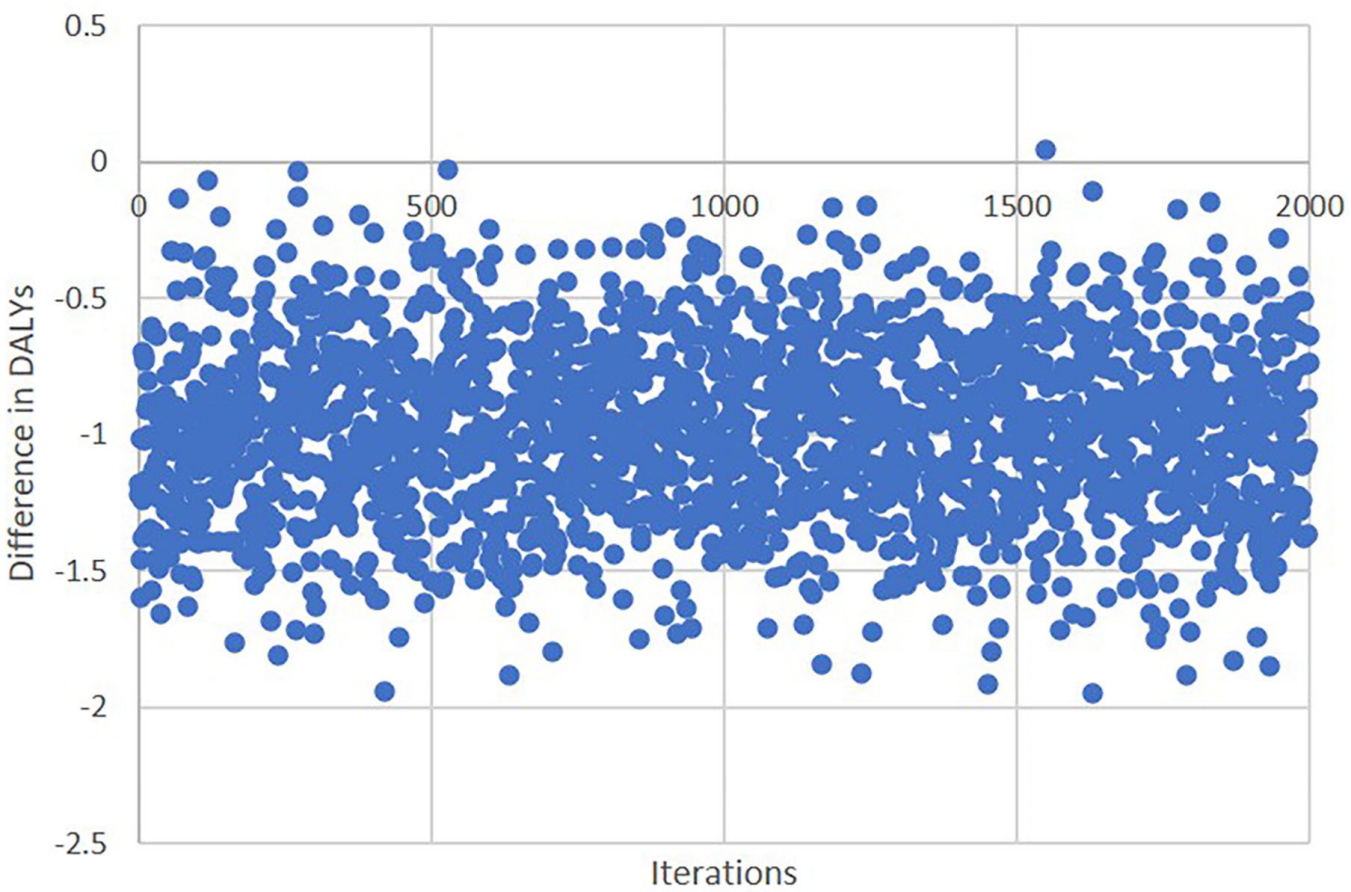
Sensitivity analysis for difference in disability-adjusted life years from bootstrapping.

The bootstrapping revealed results consistent with the base case results from GLM analysis (Table 4). The differences in INSPIRE DALYs between the treatment groups were −0.97 (95% CI: −1.60 to −0.34, *p* = 0.002) in the unadjusted analysis and −0.55 (95% CI: −0.88 to −0.23, *p* = 0.001) in the adjusted analysis (Figure 3).

**Table 4:** Sensitivity analysis from bootstrapping INSPIRE.

	**Unadjusted**		**Adjusted**	
	**INSPIRE between-group**	***p*-value**	**INSPIRE between-group**	***p*-value**
	**difference**		**difference**	
YLL	−0.63 (95% CI: −1.12, −0.15)	0.011	−0.36 (95% CI: −0.66, −0.06)	0.018
YLD	−0.27 (95% CI: −0.56, 0.01)	0.063	−0.16 (95% CI: −0.32, −0.00)	0.046
DALY	−0.97 (95% CI: −1.60, −0.34)	0.002	−0.55 (95% CI: −0.88, −0.23)	0.001

*YLL, years of life lost; YLD, years lived with a disability; DALY, disability-adjusted life year*.

## Discussion

This study demonstrated that the clinical outcomes of LVO patients who undergo EVT LVO in Australia (i.e., INSPIRE) resulted in less than half of the DALYs saved compared with those selected for enrollment in EXTEND-IA. However, EVT still resulted in significantly better DALY outcomes than those patients treated with/without thrombolysis in the INSPIRE. The saved INSPIRE DALYs from EVT were greater in the younger age group due to their longer life expectancy. The sensitivity analysis using a bootstrapping technique yielded similar results as the base case analysis with a slightly smaller difference in saved DALYs.

The smaller between-group difference in DALYs saved after EVT in INSPIRE compared with EXTEND-IA occurred despite generally comparable baseline characteristics including age, occlusion site, baseline NIHSS, ischemic core, and perfusion lesion volume (6). The primary reason for the discrepancy is that a higher proportion of patients randomized to EVT in the EXTEND-IA trial achieved much better functional outcomes proportionally at 3 months than observed from the current registry data: over 72% of patients achieved a mRS score ≤2, whereas this proportion was only 48% in the real-world data (6), probably due to offering EVT to extended time window patients (i.e., around 17% of patients received EVT beyond 6 h of the normal time window) or patients without a target mismatch (i.e., ~12% of EVT patients did not have a target mismatch, and 6% of patients had a core volume >70 ml) in INSPIRE. Indeed, the outcomes achieved in EXTEND-IA are exceptionally high by any standard and indicate rigorous adherence to computed tomography perfusion (CTP) mismatch criteria, as opposed to MR CLEAN, for example, which did not use CTP selection, and the corresponding 3-month mRS 0–2 was 32.6% (3). It is worth noting that the key driver for the post-stroke survival and DALYs avoided was the HR of mortality according to the 3-month mRS score (i.e., higher mRS score is associated with higher HR in terms of overall mortality). The substantial difference in the proportion of patients with favorable mRS score (≤2) between the RCT and the real-world post-EVT (i.e., patients with severe disability are more likely to die in the following years than those with mild disability) resulted in the difference in the YLL and subsequently led to increased YLD due to the higher disability weight associated with poorer mRS outcome.

The survival analysis and 3-month outcomes did not show that INSPIRE EVT was significantly better than the no EVT treatment group. However, when restricting the analysis to patients with ICA or M1 occlusion, both analyses suggested that INSPIRE EVT was associated with better probability of survival and mRS outcome. One possible explanation for this is that the non-EVT patients from INSPIRE had the highest proportion of M2 occlusions (21%), whereas INSPIRE EVT patients had the lowest proportion of M2 occlusions (5.6%), in comparison with the proportions from EXTEND-IA (between 11 and 17%). Patients with M2 occlusion inherently have a better prognosis than patients with more proximal occlusion (25). The high proportion of M2 occlusion patients in the no EVT group from INSPIRE probably contributed to the non-significant difference in both survival and 3-month mRS outcomes for EVT vs no EVT in INSPIRE.

This study also showed that younger patients generated greater DALY savings regardless of the treatment received; this is echoed by the significant economic burden of stroke due to young patients primarily due to productivity losses (26–28). The substantial burden of young stroke warrants national policy consideration and a targeted campaign to raise awareness of the disease in this young age group.

Quantification of DALYs from real-life data bears two policy implications: (i) the DALY estimation based on tightly controlled research trial data is not directly generalizable to everyday clinical practice. In the real-world, the selection of patients who receive EVT may not be exactly the same as the eligibility criteria for the trials, which leads to the variation in the functional outcomes. (ii) Resource allocation, guideline formulation, and priority setting in stroke treatment and prevention should preferably be based on real-world data given the potential risk of overestimation of intervention benefit from the trials. In addition, using the incremental cost due to EVT from EXTEND-IA and the DALY reduction between the EVT and no EVT groups from INSPIRE, it was estimated that the incremental cost effectiveness ratio (ICER) was A$8,838 (95% CI: $5,454–$22,726). This result suggests that with the uncertainty considered in the economic analysis, the EVT procedure may not necessarily be cost-effective in some developing countries (i.e., the upper bound of the ICER may well exceed the willingness to pay per DALY threshold).

Our study has several key strengths. Firstly, the analysis was based on routine clinical practice registry data for stroke including both EVT and intravenous alteplase treatment over the past 5 years from most of the states/territories in Australia. However, there is no doubt that this still represents a minority of patients treated with EVT in all Australian centers over the study period. Secondly, INSPIRE participants not only include those who received EVT meeting CTP mismatch criteria from trials, such as EXTEND-IA, but also incorporate a minority of patients outside of these criteria who were treated with EVT at clinicians' discretion. Thirdly, individual patient-level data facilitated the analysis that adjusted for baseline characteristics (age, gender, baseline NIHSS, baseline penumbra volume, and time from stroke onset to hospital arrival) to accurately estimate the difference in DALYs avoided between the two groups. This study, however, is not without limitations. Any recurrent stroke and other cardiovascular events beyond 3 months were not simulated in the long-term when estimating life expectancy. Given these events would impact on the patients from both treatment groups (although it could be argued that patients with better mRS outcome would have lower risk of experiencing follow-up adverse cardiovascular events), this is considered to not favor EVT. Second, the differences in patient characteristics between the INSPIRE and the EXTEND-IA including more patients with large infarct core, absence of target mismatch, and longer time from stroke onset to hospital arrival in INSPIRE may restrict comparability. However, the INSPIRE group represents the experience of EVT and standard care without EVT in the real-world where differences in characteristics do exist for patients from both treatment groups, compared with the two randomized groups in EXTEND-IA. Lastly, we are not able to comment on unmeasured patient factors (i.e., premorbid mRS that was not mandated in the INSPIRE) that may have excluded a patient from a trial, such as EXTEND-IA, but led to their inclusion in INSPIRE as part of everyday practice. While this point leads to the strength of this analysis on the implementation of the evidence, we are unable to identify the key drivers of these influencing factors.

The recently published DIRECT-MT and SKIP trials assessed whether EVT alone was inferior to the alteplase prior to EVT, with the former demonstrating the non-inferiority (mRS shift, adjusted OR: 1.07, 95% CI: 0.81–1.40, *p* = 0.04) (29–31). It would be of research interest to compare the long-term health gains from bridging alteplase in real-world from INSPIRE vs. DIRECT-MT/SKIP RCTs. However, we expect the cost implications of such direct treatment, where there is limited clinical change, to be minimal since the majority of costs arise from the long-term care of patients.

## Conclusions

EVT was associated with significant savings in DALYs compared with usual care/IVT in real-world Australian practice, but the benefit was smaller than that seen in EXTEND-IA. The incomplete translation of the EXTEND-IA results into real-world practice is likely related to multiple factors including patient selection.

## Data Availability Statement

The datasets presented in this article are not readily available because of ethics consideration. Requests to access the datasets should be directed to Associate Professor Andrew Bivard, abivard@unimelb.edu.au.

## Ethics Statement

The studies involving human participants were reviewed and approved by The Hunter New England Area Health Service Human Research Ethics Committee. The patients/participants provided their written informed consent to participate in this study.

## Author Contributions

LG and AB conceived and designed the study. LG, ET, and MM conducted the analysis. LG drafted the manuscript. MP, NS, CL, KB, TK, BY, CC, LL, PC, and AB contributed to the data collection and provided important input to the study. All authors consent to submit the paper.

## Conflict of Interest

The authors declare that the research was conducted in the absence of any commercial or financial relationships that could be construed as a potential conflict of interest.
