# Supplementary material for: Reduced Impact of Endovascular Thrombectomy on Disability in Real-World Practice, Relative to Randomized Controlled Trial Evidence in Australia
Source: Front Neurol. 2020 Dec 8;11:593238. doi: 10.3389/fneur.2020.593238 (PMC7753020; doi:10.3389/fneur.2020.593238)
Supplement: Supplementary file 1 [file Table_1.PDF]

Supplementary Materials

Supplementary Tables

Supplementary Table 1. 3-month mRS outcomes for patients with ICA or M1 occlusions

	**EVT (N=168)**	**No EVT (N=461)**
mRS0	25 (14.9%)	65 (14.1%)
mRS1	39 (23.2%)	69 (15.0%)
mRS2	19 (11.3%)	49 (10.6%)
mRS3	37 (22.0%)	59 (12.8%)
mRS4	11 (6.5%)	69 (15.0%)
mRS5	8 (4.8%)	54 (11.7%)
mRS6	29 (17.3%)	96 (20.8%)

ICA: internal carotid artery; EVT: endovascular thrombectomy; mRS: modified Rankin scal

Supplementary Table 2. Results of Cox regression model

	Hazard Ratio	Std. Err.	p-value	95% confidence Interval	
				Lower bound	Upper bound
**EVT (Yes/No)**	0.917	0.074	0.287	0.783	1.075
**Onset Age**	1.194	0.011	0.000	1.173	1.216
**Gender**	1.606	0.115	0.000	1.396	1.847
**Baseline NIHSS**	1.000	0.000	0.146	0.999	1.000
**Baseline infarct core (ml)**	1.011	0.001	0.000	1.008	1.014
**Penumbra volume (ml)**	1.000	0.001	0.551	0.999	1.002

EVT: endovascular thrombectomy; NIHSS: National Institute of Health Stroke Severity; Std.Err: standard error

Supplementary Figures

Supplementary Figure 1. Onset age distribution from the two treatment groups

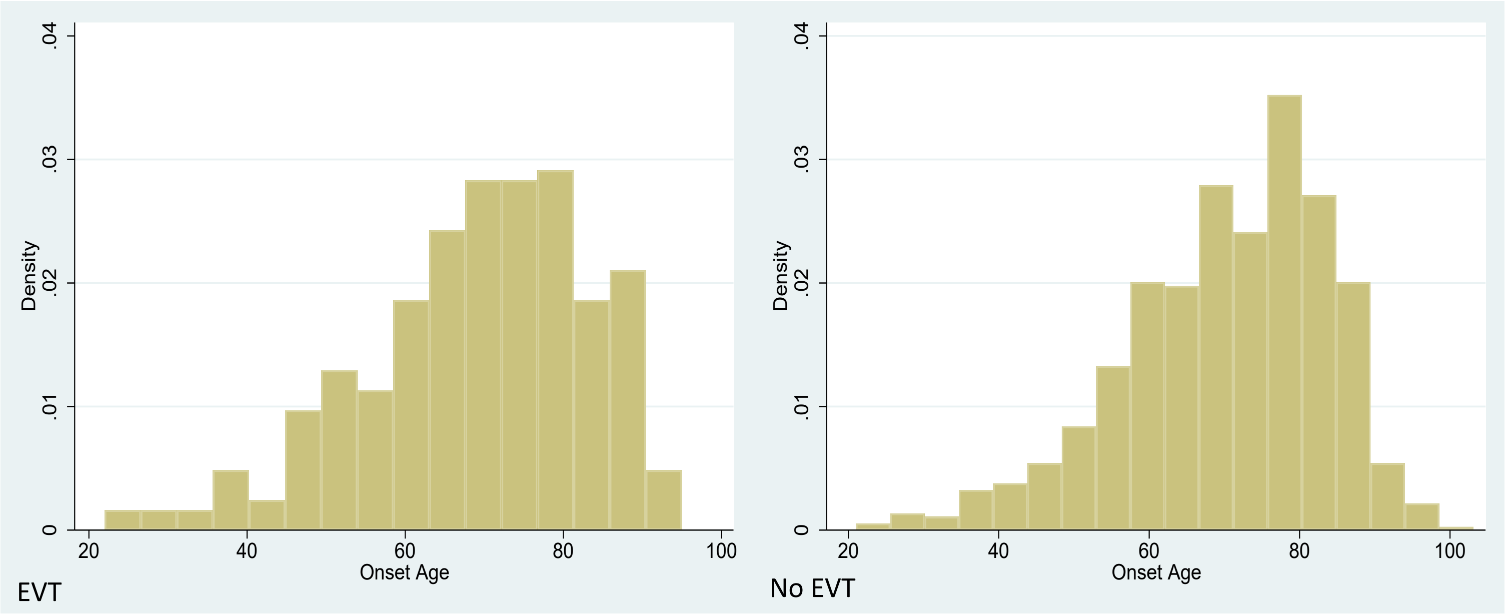

Supplementary Figure 2. Scores on the Modified Rankin Scale at 3 months_ INSPIRE registry

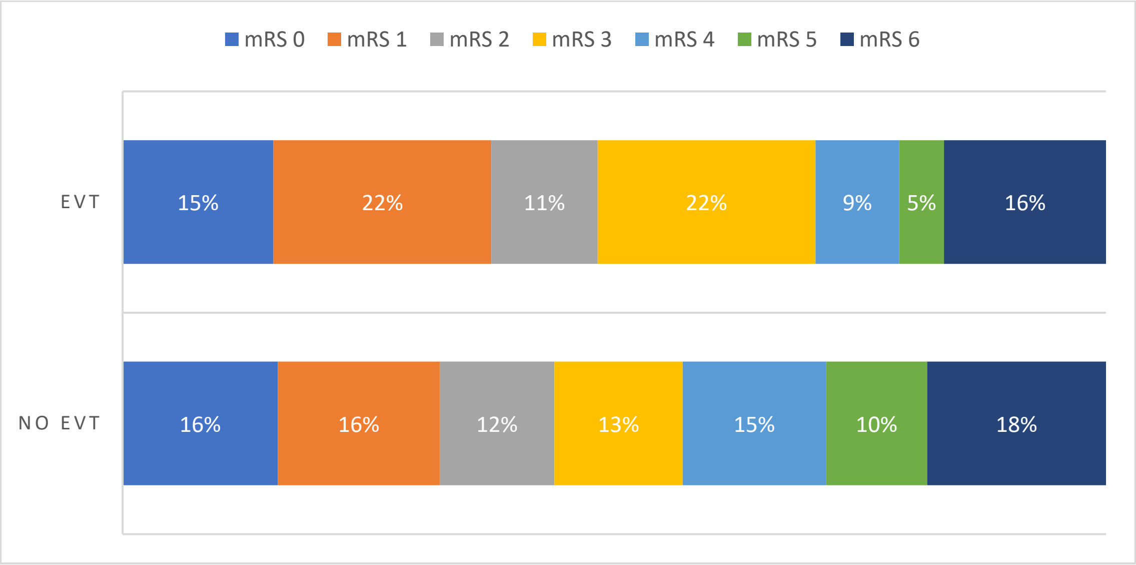

Supplementary Figure 3. Projected survival modelled over 18 years (using extrapolation from 3 month modified Rankin scale)_ subgroup analysis in patients with ICA or M1 occlusion

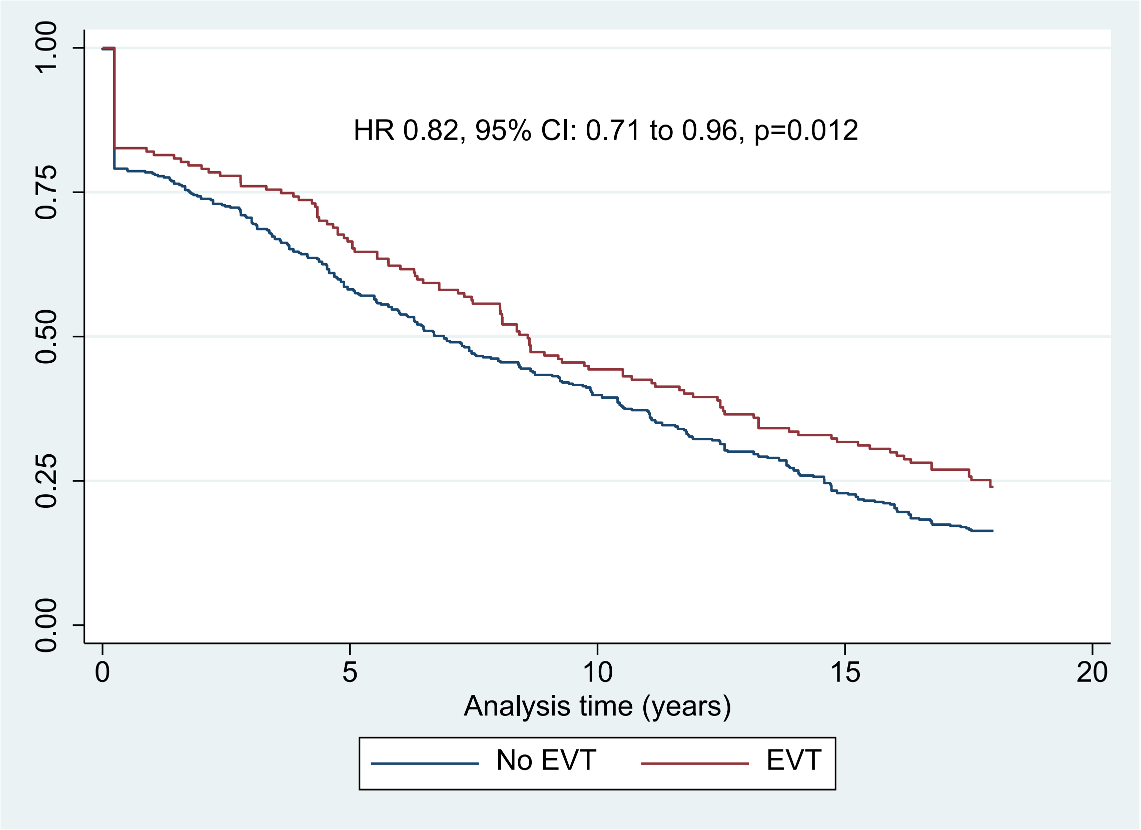

ICA: internal carotid artery; EVT: endovascular thrombectomy; HR: hazard ratio; CI: confidence interval; the hazard ratio is estimated from Cox regression model.

Supplementary Figure 4. Distribution of disability adjusted life years by treatment groups

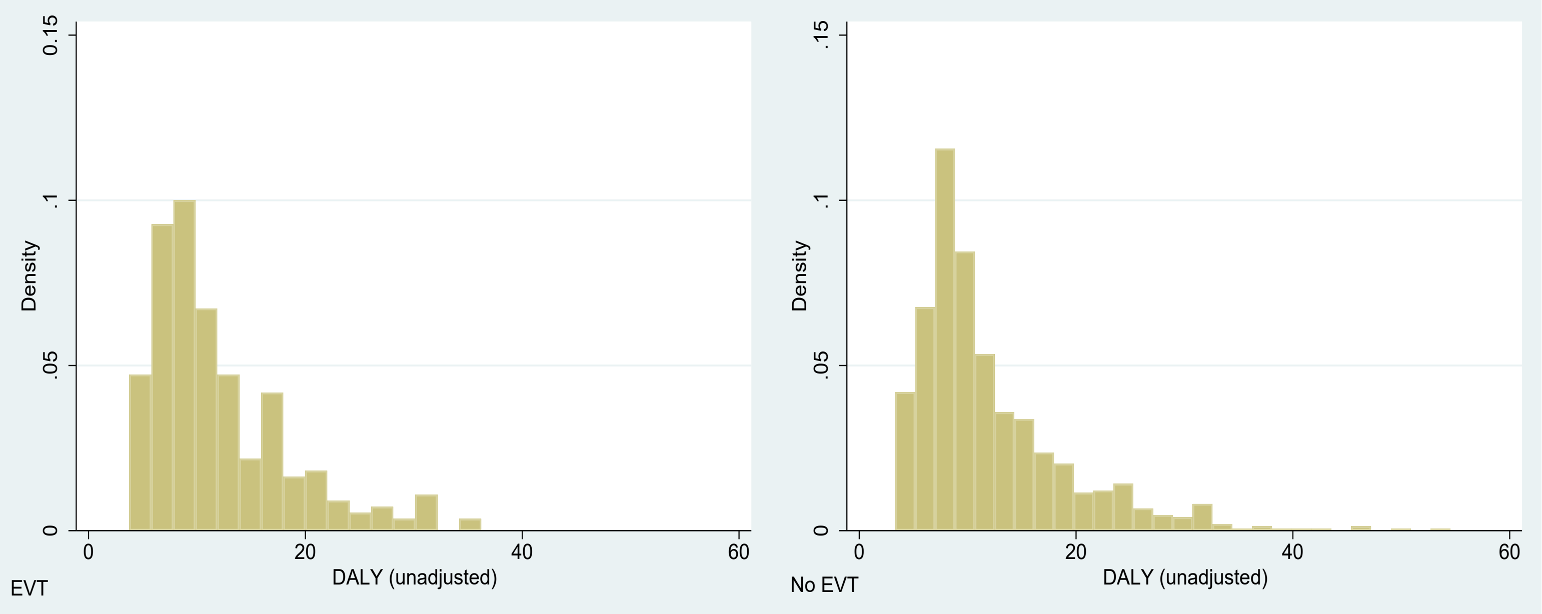

Supplementary material for calculating DALYs

Disability adjusted life years (DALYs) are calculated as the summation of the years of life lost (YLL) and years of life lived with disability (YLD). The YLL and YLD are determined by the following formulae [26]:

$$\mathrm{YLLs}\,[\mathrm{r},\mathrm{K},\beta] = \frac{KCe^{ra}}{(r+\beta)^2}\{e^{-(r+\beta)(L+a)}[-(r+\beta)(L+a)-1] - e^{-(r+\beta)a}[-(r+\beta)a-1]\} + \frac{(1-K)}{r}(1-e^{-rL})$$

Where K= age weighting modulation factor; C= constant; r= discount rate; a= age of death; β= parameter from the age weighting function; L= standard expectation of life at age a

$$\mathrm{YLDs}\,[\mathrm{r},\mathrm{K},\beta] = D\frac{KCe^{rAo}}{(r+\beta)^2}\{e^{-(r+\beta)(Ld+Ao)}[-(r+\beta)(Ld+Ao)-1] - e^{-(r+\beta)Ao}[-(r+\beta)Ao-1]\} + \frac{(1-K)}{r}(1-e^{-rLd})$$

Where K= age weighting modulation factor; C= constant; r= discount rate; Ao= age at disability onset; β= parameter from the age weighting function; Ld= years lived with disability; D= disability weight

In the base case of our analyses, DALYs were not adjusted, where DALY [0, 0, 0]. With this method, note that YLL = L, and YLD = D * Ld. For our sensitivity analyses, DALYs were discounted at 3% and age-weighted, where DALY [0.03, 1, 0.04]. The calculations for YLL and YLD were performed using the equations described above.

To account for the increased all-cause mortality in the post-stroke population, life tables were constructed for each health state (defined by mRS score at 3-months). These were adjusted from the 2016-2018 general Australian population life table by multiplying the expected death rate for each age and gender with reported hazard ratios (1.53, 1.52, 2.17, 3.18, 4.55, 6.55 for mRS 0 – 5 respectively) [16]. Life expectancy was recalculated using the formulae provided by the Australian Bureau of Statistics (ABS).

For survivors, disability weights of 0.000, 0.053, 0.228, 0.353, 0.691, 0.998 obtained from Hong & Saver [18] were used for modified Rankin Scale (mRS) scores of 0 to 5 respectively.

Examples

The calculation of DALYs is demonstrated in the following 2 examples.

Example 1: a 65 year old male has a fatal stroke

- Base case:

YLL= L, L=19.89 (remaining life expectancy from the 2016-2018 general Australian life table)
YLD= 0 (there is no duration of disability)
Therefore, DALYs= 19.89 + 0 = 19.89

- Sensitivity analyses:

$\text{YLLs}\ [0.03, 1, 0.04] = \frac{1*0.1658e^{(0.03*65)}}{(0.03+0.04)^2}\{e^{-(0.03+0.04)(19.89+65)}[-(0.03 + 0.04)(19.89 + 65) - 1] - e^{-(0.03+0.04)65}[-(0.03 + 0.04)65 - 1]\} + \frac{(1-1)}{0.03}(1 - e^{-0.03*19.89}) = 9.61$

YLD= 0

Therefore, DALYs= 9.61 + 0 = 9.61

Example 2: a 65 year old male has a stroke and survives with a mRS score at 3-months of 3.

- Base case:

The remaining life expectancy post stroke is 11.93 years (from the adjusted life table incorporating the hazard ratio of 3.18), and the patient is expected to live to 76.93 (65+11.93).

YLL= 10.99 (remaining life expectancy from the 2016 -2018 general Australian life table at age of premature death (76.93))

YLD= 0.353 * 11.93 = 4.21

Therefore, DALYs= 10.99 + 4.21 = 15.20

- Sensitivity analyses:

$\text{YLLs}\ [0.03, 1, 0.04] = \frac{1*0.1658e^{(0.03*77)}}{(0.03+0.04)^2}\{e^{-(0.03+0.04)(10.99+77)}[-(0.03 + 0.04)(10.99 + 77) - 1] - e^{-(0.03+0.04)77}[-(0.03 + 0.04)77 - 1]\} + \frac{(1-1)}{0.03}(1 - e^{-0.03*10.99}) = 4.78$

As death shall occur in the future (11.93 years), this is discounted:

$YLL = 4.78\text{e}^{-0.03*11.93}$ = 3.34

$\text{YLDs}\ [0.03, 1, 0.04] = 0.353\,\frac{1*0.1658e^{0.03*65}}{(0.03+0.04)^2}\{e^{-(0.03+0.04)(11.93+65)}[-(0.03 + 0.04)(11.93 + 65) - 1] - e^{-(0.03+0.04)65}[-(0.03 + 0.04)65 - 1]\} + \frac{(1-1)}{0.03}(1 - e^{-0.03*11.93}) = 2.47$

Therefore DALYs= 3.34 + 2.47 = 5.81
